# Response to Comment on 26 cm Fall Caught on Video Causing Subdural Hemorrhages and Extensive Retinal Hemorrhages in an 8‐Month‐Old Infant

**DOI:** 10.1002/ccr3.70702

**Published:** 2025-10-01

**Authors:** Chris Brook, Waney Squier, Julie Mack

**Affiliations:** ^1^ Universidad de La Laguna La Laguna Tenerife Spain; ^2^ Previously Department of Neuropathology John Radcliffe Hospital Oxford UK; ^3^ Radiology Penn State Hershey Medical Center Hershey Pennsylvania USA

We thank Dr. Shouldice and Dr. Smith for their interest in our case report, “26 cm Fall Caught on Video Causing Subdural Hemorrhages and Extensive Retinal Hemorrhages in an 8‐Month‐Old Infant” [1].

In their comment [2], Shouldice and Smith assert that “existing literature… supports the predominant opinion that subdural hemorrhages and retinal hemorrhages are highly associated with inflicted injury.” However, this literature has been criticized for its poor methodology, including a lack of appropriate reference standards to classify injuries as inflicted, and frequent use of circular reasoning [3, 4, 5]. An alternative interpretation that such findings are markers of the degree of intracranial pathology is supported by the data [4, 6, 7].

The authors ask a series of questions to which we respond:

1. Could the authors clarify the timing of the fall from the trampoline, relative to the timing of the twitching symptoms and the date of the head circumference?

The head circumference had grown from the 48th percentile at birth to the 99th percentile at 4 months of age, 4 months prior to the fall from the trampoline, which occurred a few days prior to the fall at the creche that was caught on video. The previous twitching began around 7 weeks earlier. The twitching occurred over a 3‐week period and was assessed 1 month prior to the creche fall and considered not concerning by the attending doctors.

2 The authors describe symptoms of vomiting and poor eating 2 days prior to the fall from the couch. We note that these can be symptoms of traumatic head injury, in addition to other possible etiologies, such as common viral illnesses. Could the authors clarify the clinical assessment regarding the cause of these symptoms, including history, physical examination, or other testing?

No medical assessment was performed because the symptoms were non‐specific, common in otherwise healthy children, and resolved without intervention before the fall. While vomiting and poor eating can occur with head trauma, they are more often attributable to benign causes, such as viral illnesses, and were not considered serious at the time.

3 Could the authors clarify whether a bleeding disorder work‐up was completed and, if so, it would be helpful to understand which tests were completed and the results?

Glutaric aciduria type 1 was excluded due to a normal urine amino acid profile. The full blood count showed a slightly elevated white count (13.9 × 10^9^/L), normal hemoglobin (105 g/L), and normal platelets (541 × 10^3^). Basic coagulation studies (PT, INR, APTT) were normal. An extended coagulation profile showed normal thrombin time, fibrinogen, factor XI and VII assays, and a normal factor XII screen. Factor II, VII, and XI levels were elevated. No abnormalities were detected on von Willebrand studies. Platelet function aggregation studies showed high levels, which the hematology team advised were not significant.

The report of child protection concluded that “investigations for possible underlying conditions predisposing to subdural hemorrhage did not reveal any evidence of metabolic or bleeding conditions which would predispose [infant name] to the development of subdural or retinal hemorrhages.”

4 Were the subdural hemorrhages all supratentorial, or were there also infratentorial hemorrhages? Would it be possible to share an axial or coronal MRI FLAIR image, which may provide improved visualization of the subdural and subarachnoid spaces?

While we did not obtain a coronal FLAIR image, we did obtain an axial FLAIR MRI, which clearly demonstrates the subdural collections as bilateral, involving frontal, parietal, occipital, and middle cranial fossa dura, including extending along a portion of the midline dural fold (falx). As noted in the case report, the collections are holohemispheric, involving both cerebral hemispheres reflecting the ability of subdural blood to track widely in the subdural compartment. They do not involve the infratentorial dura.

For additional context, the sagittal T1 MRI image included in the case report clearly delineates the supratentorial subdural collection as distinct from the subarachnoid cerebral spinal fluid (CSF) which is visible as a darker signal dipping in between the brain sulci.

We appreciate the opportunity to clarify this point and are happy to provide an annotated image highlighting these findings for further clarity.

5 Was any intervention required for increased intracranial pressure? Was there an indication of cerebral edema on the CT scan completed on the day of the described event? It would also be helpful to understand the clinical course of symptoms following the day of the fall from the couch and during hospitalization.

There was no indication of cerebral edema on the CT scan completed on the day of the described event. There was no clinical or radiographic evidence of increased intracranial pressure. The infant continued to experience projectile vomiting for several days and remained in the hospital for 6 days. No intervention was required, as the symptoms resolved during this time.

*Long‐term follow‐up can likewise be informative*. *In the months following the injury, what was the child's neurodevelopmental status and follow‐up head imaging?*

The child is now 4 years old and continues to develop normally. Follow‐up head imaging has not been required.

## Author Contributions

**Chris Brook:** conceptualization, writing – original draft. **Waney Squier:** conceptualization, investigation, writing – review and editing. **Julie Mack:** conceptualization, investigation, writing – review and editing.

## Ethics Statement

The parent gave consent for presenting medical findings in the case of her son. This consent is found in the original Case Report files.

## Data Availability

The authors have nothing to report.
